# Supplementary material for: Inflammasome Activation in Retinal Pigment Epithelium from Human Donors with Age-Related Macular Degeneration
Source: Cells. 2022 Jun 30;11(13):2075. doi: 10.3390/cells11132075 (PMC9265516; doi:10.3390/cells11132075)
Supplement: Supplementary file 1 [file cells-11-02075-s001.zip › cells-1720504-supplementary.pdf]

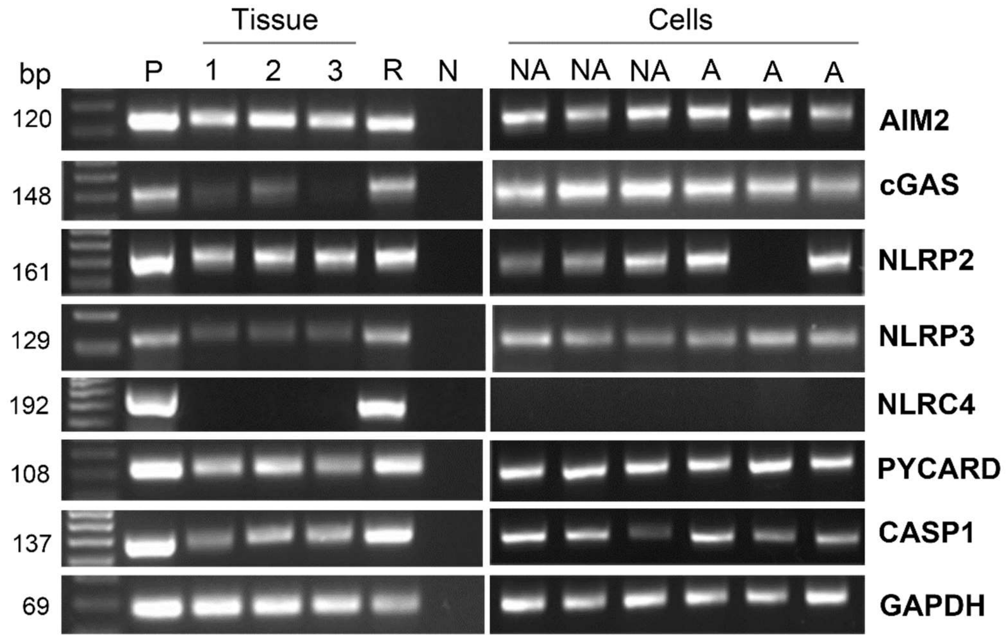

Figure S1. Inflammasome gene expression in haRPE tissue and cell cultures. (A) Gel images showing PCR products of inflammasome components in haRPE tissue (1 = MGS4, 87 year old female; 2 = MGS2, 87 year old male; 3 = MGS1, 56 year old male) and cell cultures from donors with (A) and without AMD (NA). 50bp ladder was used as reference for PCR product size. P = positive control, N = negative control (no DNA), R = retina. GAPDH is loading control.

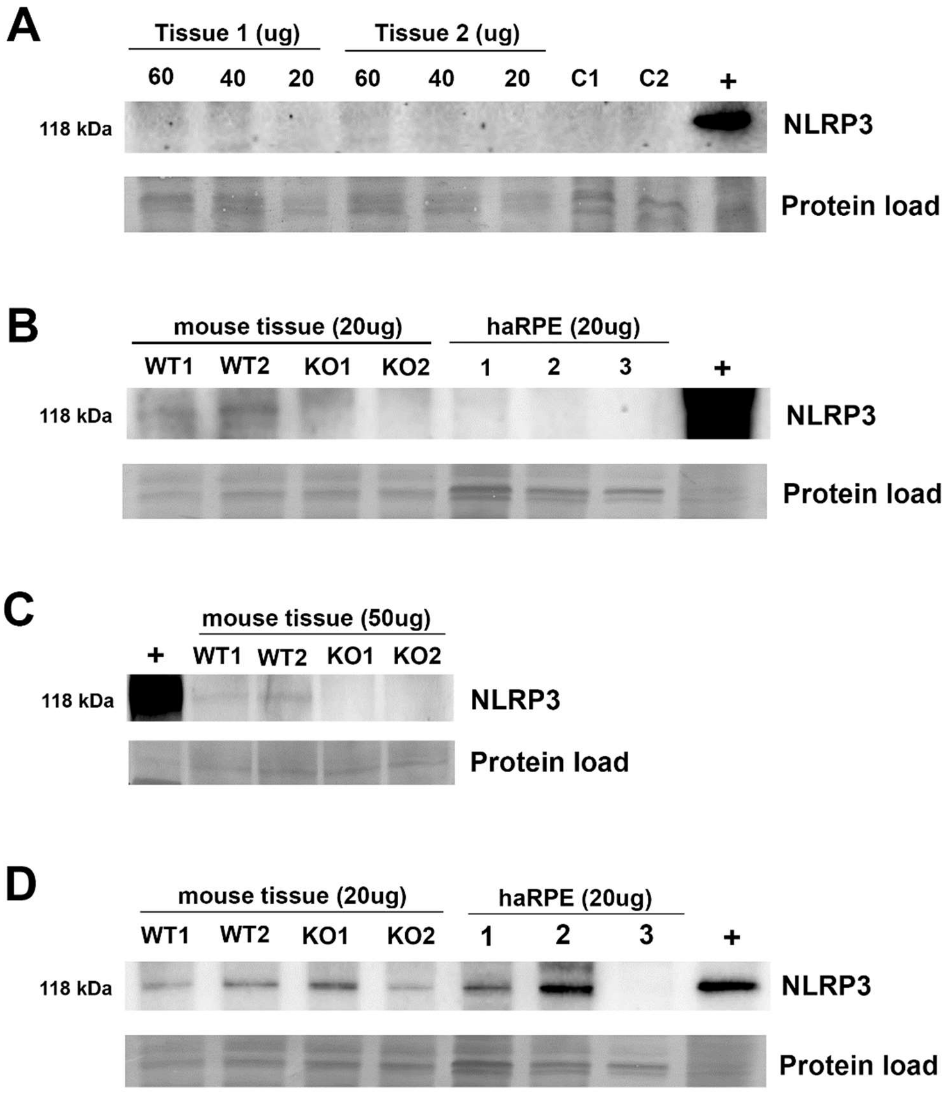

Figure S2. Checking for NLRP3 by Western blot. (A) Varying amounts RPE tissue lysates were run on Western blot to check for NLRP3 content using Cell Signaling antibody (#15101). C1 = Daudi cell lysate (x Ug), C2 = HeLa cell lysate (x ug). **(B)** Protein lysates (20µg/well) of wild-type mouse (WT) and NLRP3-/- mouse (KO) tissue was run on a Western blot along with haRPE cell lysates (1-3) to check for NLRP3 content using Cell Signaling antibody. **(C)** A larger amount (50µg) of mouse tissue lysates were run on Western Blot to confirm NLRP3 Cell Signaling antibody specificity. **(D)** Mouse tissue and haRPE cell lysates were run on Western blot to show the non-specificity of NLRP3 antibody from abcam (ab214185). Positive control (+) = THP1 cell lysate (x ug). Blots were stained with Coomassie Blue to show protein load.

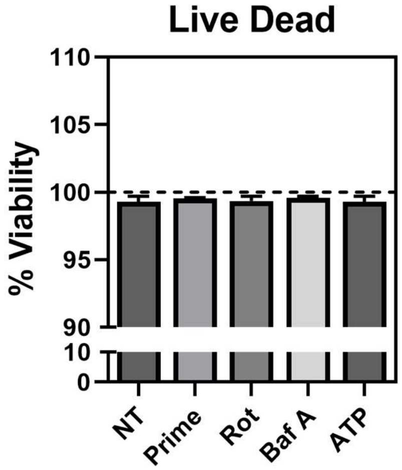

Figure S3. Measuring cell viability after inflammasome activation. Live/Dead Assay was used to measure cell viability in confluent haRPE cells (n=3) after inflammasome activation. Data was normalized to lysis (0% viability). Graph shows mean ± SEM. Dashed line = 100% viability. NT = no treatment, prime = IL-1α + LPS only, Rot = rotenone, Baf A = Bafilomycin A.

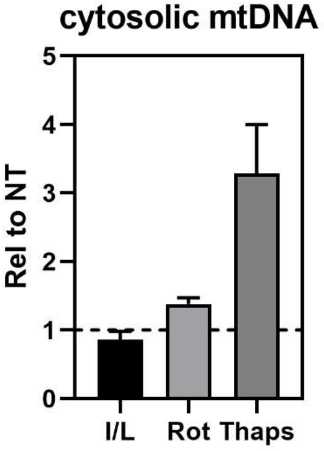

Figure S4: Determining the amount of mtDNA in the cytosol. mtDNA was extracted from the cytosol and quantified using qPCR. Data shown is fold change relative to NT =1 (dashed line). NT = no treatment, I/L = IL-1α + LPS only (prime), Rot = rotenone, Thaps = Thapsigargin (positive control for mtDNA release into the cytosol).

Table S1. Donor Demographics – haRPE tissue samples used in Mass Spec[A]

Disease State[B]/ Genotype[C]	Sample[D] (n)	Sex Male (n)	Sex Female (n)	Age[E] (Mean ± SD)	Time[F] (Mean ± SD)	Cause of Death[G] (n)
**No AMD** CFH CT	32	16	16	77 ± 10.6	19 ± 4.2	Cancer (6), CVA (2), Dementia (1), Heart failure (4), Hemorrhage (3), Hypoxia (1), Multi-system failure (2), Natural causes (1), Pneumonia (3), Pulmonary fibrosis (1), Respiratory failure (2), Sepsis (5), TBI (1)
**AMD** CFH CT	45	22	23	80 ± 10.4	19 ± 4.4	AAA (1), ALS (1), Alzheimer's disease (3), CAD (1), Cancer (17), CVA (2), ESRD (1), Heart failure (3), Hemorrhage (1), High potassium (1), Natural causes (1), PE (1), Pneumonia (4), Pulmonary fibrosis (1), Respiratory failure (1), Sepsis (5), TBI (1)

AAA = abdominal aortic aneurysm; ALS = amyotrophic lateral sclerosis; CAD = coronary artery disease; CVA = cerebrovascular accident; ESRD = end-stage renal disease; PE = pulmonary embolism; TBI = traumatic brain injury.

[A] Information supplied by Lions Gift of Sight (St. Paul, MN).

[B] Minnesota Grading System (MGS) was used to evaluate the stage of AMD in eye bank eyes (Olsen and Feng, 2004). No AMD = MGS1; AMD = MGS2

[C] Complement Factor H (CFH) genotype for rs1061170; low risk = TT, high risk = CT and CC. Note: all donors were high risk = CT.

[D] Sample number indicates the total donors used in the current study for each disease state.

[E] Age of donor (in years).

[F] The time from death to RPE harvest in hours.

[G] The number of donors for each cause of death is indicated in parentheses.

Table S2. Donor Characteristics and Clinical Information – haRPE cell cultures[A]

Disease State[B]/ Genotype[C]	Sample[D] (n)	Sex Male (n)	Sex Female (n)	Age[E] (Mean ± SD)	Time[F] (Mean ± SD)	Cause of Death[G] (n)
**No AMD**	23	12	11	63 ± 12.7	19 ± 4.2	AAA (1), ABI (3), ACE (3), Cancer (5), ESRD (1), Heart Failure (3), ICB/ICH (1), Pneumonia (1), PE (1), Sepsis (4)
Low risk	9	6	3	60 ± 12.5	20 ± 4.2	
High risk	14	6	8	65 ± 12.8	19 ± 4.3	
**AMD**	39	26	13	74 ± 9.5	20 ± 3.5	ACE (3), Cancer (9), Cirrhosis (1), COPD (2), CVA (5), Heart Failure (5), Pneumonia (5), PE (1), Respiratory disease (1), Sepsis (7)
Low risk	15	10	5	74 ± 10	18 ± 3.8	
High risk	24	16	8	74 ± 9.4	20 ± 4.1	

AAA = abdominal aortic aneurysm; ABI = Anoxic Brain Injury; ACE = acute myocardial event; COPD = chronic obstructive pulmonary disease; CVA = cerebrovascular accident (stroke); ESRD = end-stage renal disease; Heart failure = Myocardial infarction or cardiac arrest; ICH/ ICB = Intracerebral hemorrhage/ Intracerebral bleed; PE= pulmonary embolism.

[A] Information supplied by Lions Gift of Sight (St. Paul, MN).

[B] Minnesota Grading System (MGS) was used to evaluate the stage of AMD in eye bank eyes (Olsen and Feng, 2004). No AMD = MGS1; AMD = MGS2 and MGS3.

[C] Complement Factor H (CFH) genotype for rs1061170; low risk = TT, high risk = CT or CC.

[D] Sample number indicates the total donors used in the current study for each disease state.

[E] Age of donor (in years).

[F] The time from death to RPE harvest in hours.

[G] The number of donors for each cause of death is indicated in parentheses.

Table S3. Antibodies used in this study

Antibody		Dilution	Company	Product number
RPE65	WB	1:1000	Novus Biologicals; Centennial, CO, USA	NB100-355
CRALBP	WB	1:1000	Novus Biologicals; Centennial, CO, USA	NB100-74392
NaK ATPase	WB	1:1000	Cell Signaling; Danvers, MA, USA	23565
Keratin 18	WB	1:1000	Cell Signaling; Danvers, MA, USA	4548
NLRP3	WB	1:1000	Cell Signaling; Danvers, MA, USA	15101
NLRP3	WB	1:1000	Abcam; Cambridge, United Kingdom	Ab214185
AIM2	WB	1:1000	Cell Signaling;, Danvers, MA, USA	12948
IL-1β	WB	1:500	R & D Systems; Minneapolis, MN, USA	MAB601
Caspase-1	WB	1:1000	Cell Signaling; Danvers, MA, USA	2225
β-Actin	WB	1:5000	Santa Cruz Biotechnology; Dallas, TX, USA	Sc47778
Bestrophin	IF	1:250	Novus Biologicals; Centennial, CO, USA	NB300-164
Ezrin	IF	1:200	Cell Signaling; Danvers, MA, USA	3145
Alexa Fluor® 488 anti-mouse	IF	1:800	Life Technologies; Carlsbad, CA, USA	A21202
Alexa Fluor® 594 anti-rabbit	IF	1:800	Life Technologies; Carlsbad, CA, USA	A21207

Table S4. Primers for Real Time PCR analysis

Gene	Forward Primer	Reverse Primer
NLRP1	AATGGCCTCTGGATGAAACGT	CTCTCACAGAAGGCTCCCATG
NLRP2	ACAACTTTGAGACACCCCAAGT	TTCACCCCTGTATTCCCAATGG
NLRP3	TTCAAACGACTCCCTGGAAC	AAAGGAAGTGGACTGCGAGA
NLRC4	TGTCAAGTGAACCCTGTGACC	CTAGCACGTTCATCCTGTCGA
cGAS	CCTGCTGTAACACTTCTTAT	TTAGTCGTAGTTGCTTCCT
AIM2	AGCCTGAACAGAAACAGATGG	CTTCTTGGGTCTCAAACGTGA
PYCARD	CTGACGGATGAGCAGTACCA	CAAGTCCTTGCAGGTCCAGT
CASP1	GGGGTACAGCGTAGATGTGAA	CTTCCCGAATACCATGAGACA
IL-1β	CCACAGACCTTCCAGGAGAA	GTGATCGTACAGGTGCATCG
PPIG	CTTGTCAATGGCCAACAGAGG	GCCCATCTAAATGAGGAGTTGGT
ARBP	CGACCTGGAAGTCCAACTAC	ATCTGCTGCATCTGCTTG
GAPDH	TGCACCACCAACTGCTTCGC	GGCATGGACTGTGGTCATGAG
C3	GCTACATCATCGGGAAGGAC	CTGGCATTGTTTCTGGTTCTC
C3AR	CCTGCTGATGTGGTCTCACCT	CCTTGTGGTAGCTCAGACTCGT
CFB	CCCTATGCTGACCCCAATAC	GATTACACCAACTTGAATGAAACG
CFHv1	AACAGATTGTCTCAGTTTACCTAGC	ACCCGCCTTATACACATCCTTC
CFHv2	CTTTACCCTCTGAACTTCTGATCG	TCTGGCTGGAATAATACACACATAAC
CFI	TTGGATTCTGACTGCTGCAC	TTGTCCATATTTGGTAACGATGA
CD46	TGGCTACCTGTCTCAGATGACG	GCATCTGATAACCAAACTCGTAAG
CD55	CTGCTGGTGCTGTTGTGC	TCCTCGGGAAAACTTGTACG
CD59	GAGCCCAGGGAGGGAAAGGTT	CGAGGTTAAGGCAAAACCCTACGG
MCP1	CTCATAGCAGCCACCTTCATTC	TCACAGCTTCTTTGGGACACTT
IL6	GGTACATCCTCGACGGCATCT	GTGCCTCTTTGCTGCTTTCAC
mt191	CAGTGAAATTGACCTGCCCGTGAA	TCTTAGCATGTACTGCTCGGAGGT
18S rDNA	GGCCCTGTAATTGGAATGAGTC	CCAAGATCCAACTACGAGCTT
